# Hamman's syndrome accompanied by pneumorrhachis

**DOI:** 10.1590/0100-3984.2017.0141

**Published:** 2019

**Authors:** Andres Eduardo Cruz Guataqui, Bernardo Carvalho Muniz, Bruno Niemeyer de Freitas Ribeiro, Luis Henrique Spielmann, Miguel Angelo Milito

**Affiliations:** 1 Hospital Santa Teresa, Petrópolis, RJ, Brazil.; 2 Instituto Estadual do Cérebro Paulo Niemeyer - Departamento de Radiologia, Rio de Janeiro, RJ, Brazil.

**Figure 1:**
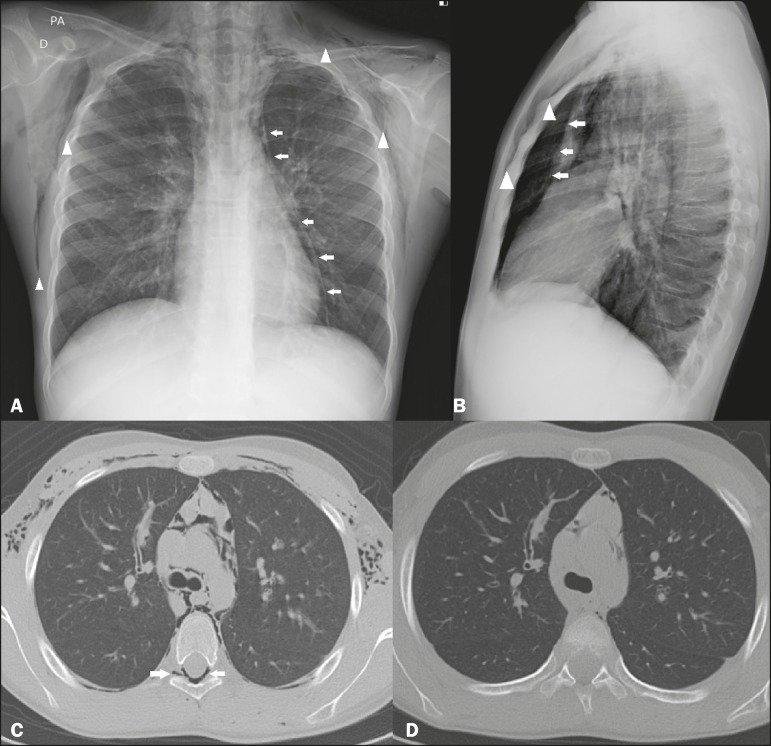
Chest X-rays, in posteroanterior and lateral views (**A** and **B**, respectively), showing pneumomediastinum (arrows) and soft tissue emphysema (arrowhead). The lateral view better identifies the air delineating the mediastinum anteriorly (arrows). CT with an intermediate window, slices at the level of the bronchial bifurcation being acquired at admission (**C**) and 72 h later (**D**), showing free air delineating the mediastinal structures, bronchi, and pulmonary vessels, as well as pneumorrhachis (arrow in **C**). Note the significant improvement of the pneumomediastinum, subcutaneous emphysema, and pneumorrhachis at 72 h after the initial CT (**D**).

Dear Editor,

An 11-year-old male patient with asthma that was being treated sporadically presented with dyspnea and acute chest pain. He had no history of recent trauma. Physical examination showed that he was afebrile and tachypneic, with crackles on palpation of the chest, neck, and axillae. A chest X-ray showed pneumomediastinum, together with bilateral subcutaneous emphysema in the soft tissues of the chest and neck (Figures 1A and 1B), findings confirmed by computed tomography (CT) of the chest, which also showed intraspinal air in the posterior aspect of the spine (pneumorrhachis), as depicted in Figure 1C. At 72 h after admission, there was clinical improvement, with a reduction in the extent of the subcutaneous emphysema and significant resorption of the initial pneumomediastinum (Figure 1D). In view of those findings, the patient was diagnosed with Hamman's syndrome accompanied by pneumorrhachis.

Spontaneous pneumomediastinum, or Hamman's syndrome, is defined as free air in the mediastinum of no apparent cause, assuming that causes such as trauma, iatrogenic complications, and infections with gas-producing bacteria have been excluded^(^1^)^. It is usually a benign, self-limiting condition that primarily affects men between 17 and 25 years of age, with an incidence of 1/30,000 hospital admissions^(^2^)^.

The pathophysiology of Hamman's syndrome is based on the Macklin effect, characterized by alveolar rupture caused by a pressure gradient between the alveoli and the pulmonary interstitium, with the consequent escape of air into the interstitium, the air then flowing toward the pulmonary hilum and mediastinum^(^3^,^^4^^)^.The main causes of spontaneous pneumomediastinum are intense physical exercise, the labor of childbirth, pulmonary barotrauma, deep dives, severe paroxysmal coughing, vomiting, asthma, a slender body type, the use of narcotics, and intense vocal effort^(^2^)^.

Clinical findings of Hamman's syndrome include chest pain, dyspnea, neck pain, and subcutaneous emphysema. One characteristic clinical sign, which can be detected on auscultation, is the presence of crackles synchronized with the beating of the heart, known as Hamman's sign or Hamman's crunch. Although Hamman's sign is highly suggestive of the condition, it is present in less than half of all cases^(^5^)^. The combination of Hamman's syndrome and pneumorrhachis is rare and is believed to be attributable to the passage of air through the posterior mediastinum to the neural foramina and epidural space^(^6^,^^7^^)^.

Chest X-rays are still the gold standard for the diagnosis of Hamman's syndrome, with a sensitivity close to 100% if posteroanterior and lateral views are obtained^(^2^)^. The main findings include linear images of gas in the mediastinum, typically extending to the neck, together with blisters or large collections of air delineating the mediastinal blood vessels, upper airways, esophagus, or heart. In cases of clinical suspicion of Hamman's syndrome, CT can be performed if the chest X-ray findings are normal or inconclusive, because it allows the anatomical localization of the air in axial slices and subsequent reconstructions. CT is also the method of choice for the diagnosis and follow-up of pneumorrhachis^(^8^)^.

The standard treatment for Hamman's syndrome is clinical observation combined with supportive measures, usually in a hospital setting. The syndrome typically resolves spontaneously after two to seven days, and recurrence is uncommon^(^5^)^.

The prevalence of Hamman's syndrome is low. Nevertheless, it should be considered in the differential diagnosis of acute chest pain, especially in young patients with subcutaneous emphysema, and the possibility of pneumorrhachis should be investigated.
